# Serum human chorionic gonadotropin ratios for the detection of etoposide, methotrexate, dactinomycin, cyclophosphamide, and vincristine resistance in high‐risk gestational trophoblastic neoplasia

**DOI:** 10.1002/hsr2.729

**Published:** 2022-07-20

**Authors:** Nathapol Sirimusika, Sathana Boonyapipat

**Affiliations:** ^1^ Unit of Gynecologic Oncology, Department of Obstetrics and Gynecology Faculty of Medicine, Prince of Songkla University, Hat Yai Songkhla Thailand

**Keywords:** drug resistance, EMA/CO, gestational trophoblastic neoplasia, human chorionic gonadotropin ratio, nomogram

## Abstract

**Aims:**

This study aimed to identify the optimal human chorionic gonadotropin (hCG) ratio in predicting etoposide, methotrexate, dactinomycin, cyclophosphamide, and vincristine resistance in women diagnosed with high‐risk gestational trophoblastic neoplasia (GTN) and to compare the chemoresistant disease detection rate by using the optimal hCG ratio and traditional criteria.

**Methods:**

Seventy‐six women with primary high‐risk GTN treated with etoposide, methotrexate, dactinomycin, cyclophosphamide, and vincristine in a tertiary‐care center were included. The hCG ratio was determined by its serum pretreatment level divided by that before each cycle of chemotherapy. The traditional criteria for chemoresistance included plateau or rising of hCG or presence of new metastasis. The optimal hCG ratio was determined using receiver operating characteristics (ROC) curve analysis.

**Results:**

Among the specificities of 90%, 92.5%, and 95%, the 90% specificity yielded the best ROC curve. At 90% specificity, the best area under curve value was at the fourth cycle with 75% sensitivity. The hCG ratio at the fourth cycle was 31.92. Using the ratio at the fourth cycle, chemoresistant disease was detected in six out of eight patients, compared to one in the traditional criteria. When combining the two diagnostic tools, the cumulative detection rate in the fourth cycle was 10/12 (83.3%) of total drug resistance. Among patients who developed drug resistance at the fourth cycle or thereafter, the use of the ratio at the fourth cycle could diagnose chemoresistance approximately two cycles earlier than that with the traditional criteria.

**Conclusions:**

A hCG ratio of <31.9 at the fourth cycle should be considered a high‐risk for etoposide, methotrexate, dactinomycin, cyclophosphamide, and vincristine resistance and may need second‐line chemotherapy. The ratio increases the detection rate of resistance to these drugs more than the traditional criteria.

## 1 INTRODUCTION

Gestational trophoblastic neoplasia (GTN) is a group of malignant neoplasms, including invasive mole, choriocarcinoma, placental‐site trophoblastic tumor (PSTT), and epithelioid trophoblastic tumor (ETT), that arises from abnormal proliferation of placental trophoblast cells during pregnancy. GTN is highly sensitive to chemotherapy with a cure rate of >90%.^1^, ^2^ The serum human chorionic gonadotropin (hCG) level is a reliable tumor marker for diagnosis and monitoring response to treatment.

Patients with GTN are classified into low‐risk (LRGTN) or high‐risk (HRGTN) using the modified World Health Organization (WHO) prognostic scoring system as adapted by the International Federation of Gynecology and Obstetrics (FIGO).^3^ HRGTN is defined by FIGO stage IV or any FIGO stage with a score of ≥7 which is unlikely to respond with single‐agent chemotherapy treatment. Therefore, the standard treatment of HRGTN is combined chemotherapy. Etoposide, methotrexate, and dactinomycin alternated weekly with cyclophosphamide and vincristine (EMA/CO) regimen is effective, less toxic, and widely used as the primary treatment in HRGTN patients.^4^, ^5^ The remission rate of EMA/CO therapy ranges from 71–86%.^6^, ^7^, ^8^, ^9^, ^10^ The patients who were refractory to EMA/CO had worse outcomes with only 43% 5‐year survival (95% confidence interval: 12–73%).^11^

The early diagnosis of chemoresistance can reduce unnecessary chemotherapy and chemotoxicity and may potentially improve the treatment outcomes. The diagnosis of EMA/CO resistance depends on a declining level of hCG. Currently, there is no consensus on the guidelines to define the criteria to determine drug resistance. A variety of tools derived from declining hCG levels were proposed to predict clinical course and response to chemotherapy such as hCG regression curve with or without a cut‐off point, hCG ratio, and hCG reduction rate.^12^, ^13^, ^14^, ^15^, ^16^

The utility hCG ratio in GTN was first introduced by Van Trommel et al.^17^ in 2008 to identify persistent trophoblastic disease.^18^ However, no study reports the use of hCG ratio to assess chemotherapy response in GTN. The current study aimed to evaluate the optimal serum hCG concentration ratios in primary HRGTN for predicting EMA/CO resistance. The secondary objective was to compare the detection rate of chemoresistant disease between optimal hCG ratio and traditional criteria.

## 2 METHODS

Following approval by the research ethics committee, the medical records of patients with primary HRGTN who received the EMA/CO chemotherapy regimen between January 1, 2002, and December 31, 2013, were retrospectively reviewed. The requirement for the acquisition of informed consent from patients was waived owing to the retrospective nature of this study. Primary HRGTN was defined as patients who had initial FIGO stage IV or any stage with WHO scoring ≥7. Patients with recurrent disease, PSTT, ETT, discontinued treatment due to drug toxicity, or lost to follow‐up and response could not be determined were excluded. Patient characteristics were reviewed for age, antecedent pregnancy, interval months from index pregnancy, size, and site of metastasis, pretreatment serum hCG level, and level before each cycle of chemotherapy, FIGO‐2000 stage, and modified WHO risk‐factor scoring system for GTN.

All primary HRGTN cases were evaluated for hCG ratios. The hCG ratio was defined as pretreatment serum hCG level divided by hCG level before each cycle of chemotherapy. The hCG ratios of each cycle were investigated by the area under the curve (AUC) from the receiver operating characteristics method (ROC) at 90%, 92.5%, and 95% specificities in predicting chemoresistance classified by the traditional criteria. The best cut‐off hCG ratio was identified. The detection of chemoresistance by using the optimal hCG ratio and the traditional criteria was compared. Cox regression analysis was performed for the univariate and multivariate analyses to identify independent risk factors associated with chemoresistance.

Complete response was defined when hCG regressed to a normal level for 3 consecutive weeks. Patients who had not achieved a complete response following the traditional criteria were considered chemoresistant: (i) plateau of hCG throughout three cycles of chemotherapy, (ii) rise in hCG of ≥10% over two cycles of chemotherapy, or (iii) presence of new metastasis.

### 2.1 Immunoassays

The serum hCG levels were determined using MODULAR ANALYTICS E170 system (Roche Diagnostics) an electrochemiluminescence immunoassay, which measures the sum of the hCG and the hCG β‐subunit. This method has been standardized against the fourth International Standard for Chorionic Gonadotropin from the National Institute for Biological Standards and Control (NIBSC) code 75/589. Serum hCG concentrations were considered normalized if <5 mIU/ml.

### 2.2 Statistical analyses

From the sample size, calculations for diagnostic tests were based on 80% sensitivity, 95% specificity, 28% prevalence of EMA/CO resistance,^10^ and 0.05 alpha level. At least 101 patients were required. Statistical calculations were performed using R software version 2.14 (The R Foundation for Statistical Computing). Differences in the numerical data between the two groups were tested nonparametrically (Mann–Whitney *U* test) and parametrically (the Student *t* test). All tests were considered statistically significant at *p* < 0.05.

### 2.3 Ethical approval

This retrospective cohort study was conducted in accordance with the principles embodied in the 1975 Helsinki Declaration, as revised in 2000, and the study protocol was approved by the Institutional Review Board and Ethics Committee of the Songklanagarind Hospital on January 8, 2013 (approval no.: 58‐016‐12‐3).

## 3 RESULTS

Based on the hospital database, 94 patients were diagnosed with GTN. Patients with recurrent GTN, PSTT, ETT and incomplete information were excluded. The remaining 76 patients were diagnosed with primary HRGTN. Of these, 64 patients achieved complete response and 12 patients (15.7%) developed EMA/CO resistance. Characteristics of the patients are shown in Table 1. Approximately 40% of HRGTN occurred after molar pregnancy. The common metastatic site was the lung (59%), and 47% of patients were in FIGO stage III. Mean WHO scores of the complete remission and chemoresistance groups were 11.5 and 13.6, respectively (*p* = 0.05). Pretreatment hCG level, FIGO stage, age, and metastatic sites were not significantly different between the complete response and chemoresistance groups.

**Table 1:** Patient characteristics

Characteristics	Complete response (*n* = 64)	Chemoresistance (*n* = 12)	*p* value
Age (years)	39 (30–47)	41.5 (36–51)	0.202
Parity			
Median (IQR)	2 (1, 3)	2 (1, 3.2)	0.674
Antecedent pregnancy			
Hydatidiform mole	27 (42.2%)	4 (33.3%)	0.563
Nonmolar abortion	16 (25.0%)	2 (16.7%)	
Term	21 (32.8%)	6 (50.0%)	
Interval from index pregnancy (months)			
<4	14 (21.9%)	0	0.131
4–6	11 (17.1%)	1 (8.3%)	
7–12	9 (14.1%)	1 (8.3%)	
>12	30 (46.9%)	10 (83.4%)	
Pretreatment hCG (ng/ml)			
Median (IQR)	266,095 (117,317–602,213)	446,694 (235,742–603,327)	0.499
Tumor size (cm)			
Mean (*SD*)	6.4 (3.5)	8.6 (3.9)	0.051
No of metastasis	9 (3,14)	6.5 (1, 13)	0.599
Metastasis sites	45 (70.3%)	10 (83.3%)	0.492
Vagina	14 (31.1%)	1 (10%)	0.255
Lung	37 (82.2%)	8 (80%)	1
Spleen, kidney	1 (2.2%)	0	1
Gastrointestine	2 (4.4%)	0	1
Liver, brain	11 (24.4%)	2 (20%)	1
FIGO stage			
I	20 (31.2%)	2 (16.7%)	0.781
II	5 (7.8%)	1 (8.3%)	
III	29 (45.3%)	7 (58.3%)	
IV	10 (15.6%)	2 (16.7%)	
WHO score (mean‐*SD*)	11.5 (3.3)	13.6 (3.4)	0.05

Abbreviations: FIGO stage, International Federation of Gynecology and Obstetrics 2000 staging; hCG, serum human chorionic gonadotropin, IQR, interquartile range, *SD*, standard deviation, WHO score, modified World health Organization risk‐factor scoring system for gestational trophoblastic neoplasia.

**Figure 1:**
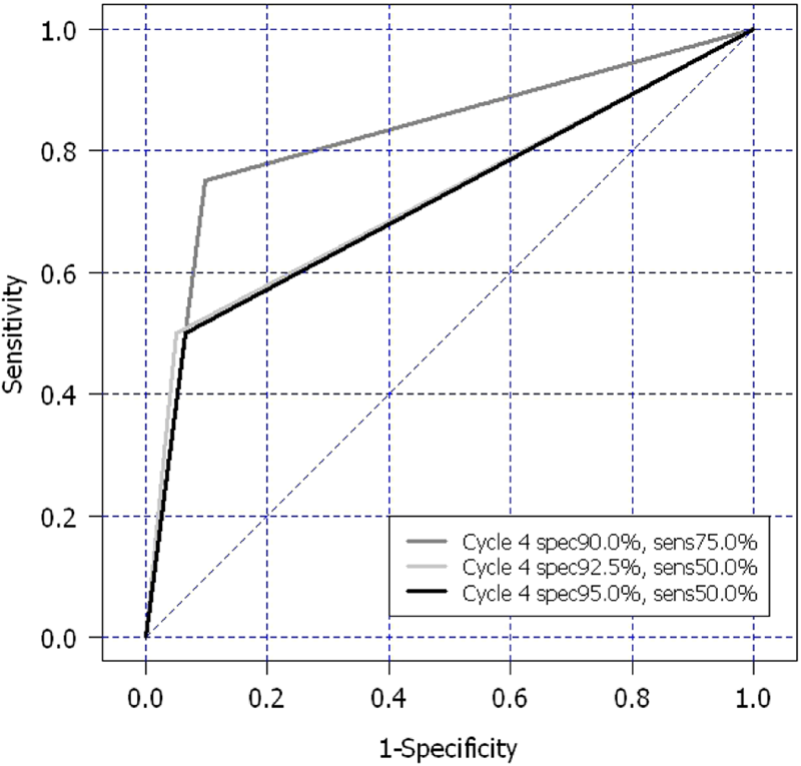
Receiver operating characteristic curve of specificity of 90%, 92.5%, and 95% at the fourth cycle of therapy (optimal cut‐point data)

Table 2 shows ROC curve analysis of hCG ratio by chemotherapy cycle for predicting EMA/CO resistance at the specificities of 95%, 92.5%, and 90%. At 90% specificity, hCG ratio at the fourth cycle of chemotherapy provided the best AUC performance (0.83) with a sensitivity of 75% for the diagnosis of chemoresistance. The optimal cut‐off hCG ratio at the fourth cycle of chemotherapy was 31.92. Figure 1 shows hCG AUCs at the specificities of 95%, 92.5%, and 90% for predicting EMA/CO resistance.

**Table 2:** Receiver operating characteristic curve analysis of hCG ratios of each cycle of treatment with EMA/CO

Specificity (%)	Cycle	Sensitivity (%)	AUC	CI (%)	Cut‐off hCG ratio
95	2	8.3	0.51	0.21–38.41	3.74
	3	41.7	0.68	15.17–72.33	13.44
	4	50.0	0.73	15.70–84.30	19.91
	5	28.6	0.62	3.67–70.96	17.89
	6	25.0	0.61	0.63–80.59	24.14
92.5	2	16.0	0.54	2.09–48.41	4.77
	3	50.0	0.71	21.09–78.91	18.16
	4	50.0	0.72	15.70–84.30	22.74
	5	42.8	0.68	9.90–81.59	28.24
	6	25.0	0.61	0.63–80.59	24.14
90	2	33.0	0.62	9.92–65.11	6.05
	3	50.0	0.71	21.09–78.91	18.74
	4	75.0	0.83	34.91–96.81	31.92
	5	43.0	0.67	9.90–81.59	41.85
	6	50.0	0.71	6.76–93.24	100.22

Abbreviations: AUC, area under the curve; CI, confidence interval; EMA/CO, etoposide, methotrexate, dactinomycin, cyclophosphamide, vincristine; hCG, human chorionic gonadotropin; ROC, receiver operating characteristic.

Among 12 HRGTN patients, eight developed drug resistance at fourth cycle or later. On using the hCG ratio at the fourth cycle, chemoresistant disease was detected in six out of eight patients, compared to one on using the traditional criteria. The traditional criteria could detect drug resistance at cycles 4 (*n* = 1), 5 (*n* = 3), 6 (*n* = 1), 8 (*n* = 2), and 9 (*n* = 1). On combining the two diagnostic tools, the cumulative detection rate at fourth cycle was 10/12 (83.3%) of total drug resistance. It was found that patients diagnosed based on the hCG ratio did not have a false negative result compared to the traditional criteria and could be diagnosed approximately two cycles (range: 0–5) earlier than that with the traditional criteria.

As shown in Table 3, univariate analysis revealed that hCG ratio at the fourth cycle and interval from index pregnancy were associated with EMA/CO resistance. We evaluated the independent prognostic factors for EMA/CO resistance using Cox proportional hazard analysis. Multivariate analysis confirmed that both hCG ratio at the fourth cycle (hazard ratio [HR]: 109.79; 95% confidence interval [CI]: 5.21, 2315.23; *p* < 0.001) and interval from index pregnancy (HR: 3.04; 95% CI: 1.29, 848.46; *p* = 0.013) were independent factors correlated with EMA/CO resistance (Table 4).

**Table 3:** Univariate and multivariate cox regression analyses for EMA/CO resistance in high‐risk gestational trophoblastic neoplasia

Variable	Univariate analysis		Multivariate analysis	
	OR (95% CI)	*p* value	OR (95% CI)	*p* value
Age				
≥40 vs. <40 years	1.13 (0.33, 3.89)	0.842	–	–
Antecedent pregnancy				
Term	2.05 (0.59, 7.12)	0.262	–	–
vs. abortion/molar				
Interval from index pregnancy (months)				
>12 vs. ≤12	5.67 (1.15, 27.94)	0.015	3.04 (1.29, 848.46)	0.013
Pretreatment hCG level				
<10000	Ref.	0.688	–	–
10,000–10,0000	0.50 (0.03, 7.54)			
>10,0000	0.55 (0.05, 5.91)			
hCG ratio at 4^th^ cycle				
<31.9 vs. ≥31.9	27.5 (4.51, 167.8)	<0.001	109.79 (5.21, 2315.23)	<0.001
Tumor size (cm)				
>5 vs. ≤5	1.46 (0.4, 5.35)	0.563	–	–
Number of metastasis				
>8 vs. ≤8	0.87 (0.22, 3.45)	0.849	–	–
Stage				
I	Ref.	0.752	–	–
II	2.00 (0.15, 26.73)			
III	2.41 (0.45, 12.84)			
IV	2.00 (0.24, 16.36)			
WHO score^a^	1.20 (0.99, 1.44)	0.052	–	–

Abbreviations: CI, confidence interval OR, odds ratio, WHO, World Health Organization.

^a^ Continuous variable.

**Table 4:** Studies of declining hCG for the prediction of chemoresistance in GTN

Study	Tool	Population	*N*	Result	Sensitivity (%)	Specificity (%)
Lertkhachonsuk^14^	Reduction rate^a^	Persistent GTD treated with single‐agent	72	Significant difference in reduction rate during 3^rd^ to 7^th^ week between the chemosensitive and chemoresistant cases	–	–
van Trommel^18^	Regression curve	LRGTN treated with MTX	79	The best cut‐off point: hCG >520 U/L at 7^th^ week	50.0	97.5
Lybol^16^	Regression curve	HRGTN treated with EMA/CO	46	90th percentile of prior single drug resistance and primary HRGTN turned to normal before 4^th^ and 8^th^ cycles, respectively.	–	–
You^19^	Population kinetic modeling	LRGTN treated with MTX	800	The best cut‐off point: hCG >20.44 mIU/ml after 3^rd^ cycle	91.0	83.0
Rattanabur^15^	Regression curve	HRGTN treated with EMA/CO	81	90th percentile of prior single drug resistance and primary HRGTN turned to normal before 2^nd^ and 9^th^ cycles, respectively. The best cut‐off point: hCG ≥118.6 mIU/ml before 5^th^ cycle	85.7	100.0
Our study	hCG Ratio^b^	HRGTN treated with EMA/CO	77	The best cut‐off point: hCG ratio <31.9 before 4^th^ cycle	75.0	90.0

Abbreviations: EMA/CO, etoposide, methotrexate, dactinomycin, cyclophosphamide, vincristine; GTD, gestational trophoblastic disease; GTN, gestational trophoblastic neoplasm; hCG, human chorionic gonadotropin; HRGTN, high‐risk gestational trophoblastic neoplasm; LRGTN, low‐risk gestational trophoblastic neoplasm; MTX, methotrexate.

^a^ Reduction rate = (median serum B‐hCG in week 1‐median serum B‐hCG in week X)/serum B‐hCG in week 1.

^b^ hCG ratio = pretreatment serum hCG level/hCG level before each cycle of chemotherapy.

## 4 DISCUSSION

Early detection of drug resistance is beneficial in decreasing the number of cycles and toxicity from chemotherapy. The present study evaluated the optimal serum hCG concentration ratios in HRGTN cases receiving EMA/CO to predict chemotherapy resistance and found that the best cut‐off point of hCG level for the diagnosis of chemoresistance is in the fourth cycle with a sensitivity and specificity of 70% and 90%, respectively. The AUC for hCG ratio to predict EMA/CO resistance demonstrates moderate discriminatory power (0.83); therefore, it has the potential utility as a diagnostic test. The hCG cut‐off ratio in the fourth cycle is an independent risk factor for EMA/CO resistance and is considered a new tool to diagnose chemoresistance with a higher detection rate than the traditional criteria.

The clinical utility of declining hCG levels that is proposed to predict chemotherapy response in GTN is shown in Table 3. van Trommel et al.^17^ first reported hCG regression curve for prediction of methotrexate resistance in LRGTN. Lertkhachonsuk et al.^14^ studied the difference of regression pattern of serum hCG levels in persistent gestational trophoblastic disease patients who were chemosensitive and chemoresistant. You et al.^19^ suggested that the best cut‐off value of hCG was after third cycle, which could predict methotrexate resistance in LRGTN.

To the best of our knowledge, this is the first study to analyse the hCG ratios in HRGTN for EMA/CO chemoresistance. Lybol et al.^16^ and Rattanaburi et al.^15^ introduced the hCG regression nomogram in HRGTN to predict EMA/CO resistance.^15^, ^16^ Rattanaburi et al.^15^ have discussed hCG regression curve for nomogram and reported that 90th percentile of the hCG level turned to normal before the eighth cycle of chemotherapy in primary HRGTN and found that a serum hCG level of >118.6 at the fifth cycle of chemotherapy predicted EMA/CO resistance with a sensitivity 85.7% and specificity of 100%.^15^ Comparing the detection rate of the hCG ratio in our study to the hCG regression curve with cut‐off value, it was found that the detection rate of hCG ratio had a lower sensitivity and specificity; however the use of hCG ratio had the advantage of being able to detect chemoresistance at one cycle earlier.

Regarding clinical applications, the detection rate and timing for the diagnosis of chemoresistance were compared to those of the traditional criteria. Using the hCG ratio at the fourth cycle of chemotherapy could detect more chemoresistant diseases than that with the traditional criteria. On combining the two diagnostic tools, the cumulative detection rate at fourth cycle increased to 83% of total drug resistance. Approximately half of the chemoresistant patients can be determined by the hCG ratio earlier than that with the traditional criteria. However, using the hCG ratio alone could not detect early chemoresistance that develops before the fourth cycle of chemotherapy which was found in approximately one‐third of total chemoresistant cases in our series.

In agreement with the previous studies, we analysed hCG ratios at high specificity levels. The previous nomograms for the prediction of resistance to methotrexate chemotherapy used upper percentiles of p95, p97.5, and p99.^18^, ^20^ The study of using population kinetic modeling of hCG measurement for detection of methotrexate resistance accepted the specificity at 83% as the best cut‐off point.^19^ Our study used the specificities of only >90% to avoid false positive rates which cause unnecessary change to more intensive chemotherapeutic regimens. We did not include GTN patients with single chemotherapy resistance due to having a difference in hCG regression curve and different prognosis.^15^

Our study has some limitations. Due to rarity of disease, the small sample size is inevitable. A prospective large sample study is needed to confirm reproducibility of the best hCG ratio and as to whether it can improve disease outcomes. Applying cut‐off values to other populations is applicable if HRGTN is defined by the same classification and if there is a similar prevalence of test positive or EMA/CO resistance rate. The EMA/CO resistance rate in our study was 15.6% which is consistent with that in previous studies on EMA/CO.^8^, ^21^, ^22^

## 5 CONCLUSION

In terms of clinical implication, we suggest using hCG ratios of <31.9 to identify drug resistance at the fourth cycle of chemotherapy. This ratio predicts the need for more intensive chemotherapy. Our findings suggest the combined use of hCG ratios and traditional criteria to improve the detection of EMA/CO resistance, possibly allowing physicians to identify chemoresistant patients in the early course of treatment. Future studies are warranted to elucidate whether the use of the hCG titer ratio leads to better outcomes.

## AUTHOR CONTRIBUTIONS

*Conceptualization*: Nathapol Sirimusika, Sathana Boonyapipat. *Data Curation*: Nathapol Sirimusika, Sathana Boonyapipat. *Formal analysis*: Nathapol Sirimusika. *Methodology*: Nathapol Sirimusika, Sathana Boonyapipat. *Supervision*: Sathana Boonyapipat. *Validation*: Nathapol Sirimusika, Sathana Boonyapipat. *Visualization*: Nathapol Sirimusika, Sathana Boonyapipat. *Writing—original draft*: Nathapol Sirimusika. *Writing—review and editing*: Sathana Boonyapipat. All authors have read and approved the final version of the manuscript. Sathana Boonyapipat had full access to all of the data in this study and takes complete responsibility for the integrity of the data and the accuracy of the data analysis.

## CONFLICT OF INTEREST

The authors declare no conflict of interest.

## TRANSPARENCY STATEMENT

The corresponding author affirms that this manuscript is an honest, accurate, and transparent account of the study being reported; that no important aspects of the study have been omitted; and that any discrepancies from the study as planned have been explained.

## Data Availability

The authors confirm that the data supporting the findings of this study are available within the article.
